# Supplementary material for: Single-cell ATAC-seq signal extraction and enhancement with SCATE
Source: Genome Biol. 2020 Jul 3;21:161. doi: 10.1186/s13059-020-02075-3 (PMC7333383; doi:10.1186/s13059-020-02075-3)
Supplement: Supplementary file 1 — Additional file 1 Table S1. A comparison between SCATE and other existing methods. [file 13059_2020_2075_MOESM1_ESM.pdf]

Additional file 1

Table S1. A comparison between SCATE and other existing methods

Method	Combine CREs	Combine cells	Adaptively tune resolution	Use public bulk data to model baseline	Use Binary or Count data	Primary goal	Reference
SCATE	√	√	√	√	C	Reconstruct activities of each individual CRE	This paper
chromVAR	√				C	Cluster cells, identify TF motifs associated with differential accessibility and variability	[12]
SCRAT	√				C	Cluster cells, identify CRE pathways associated with differential accessibility	[13]
BROCK-MAN	√				B	Summarize data by k-mers and perform principal component analysis on k-mer features to identify co-varying TFs, cluster cells	[14]
Dr.seq2		√			C	Cluster cells, identify peaks (MACS) in each cell subpopulation	[17]
Cicero		√			B	Identify correlated pairs of CREs	[18]
Scasat					B	Cluster cells, identify peaks (MACS), differential accessibility analysis	[20]
Destin					B	Cluster cells	[21]
scABC					C	Cluster cells	[22]
PRISM					B	Quantify cell-to-cell variation to identify hyper- or hypo-variable genomic features	[23]
cisTopic					B	Represent data using low-dimensional topic-cell and region-topic representation, cluster cells and CREs accordingly	[24]
