# Supplementary material for: Single-cell ATAC-seq signal extraction and enhancement with SCATE
Source: Genome Biol. 2020 Jul 3;21:161. doi: 10.1186/s13059-020-02075-3 (PMC7333383; doi:10.1186/s13059-020-02075-3)
Supplement: Supplementary file 2 — Additional file 2 Figures S1-S15. [file 13059_2020_2075_MOESM2_ESM.pdf]

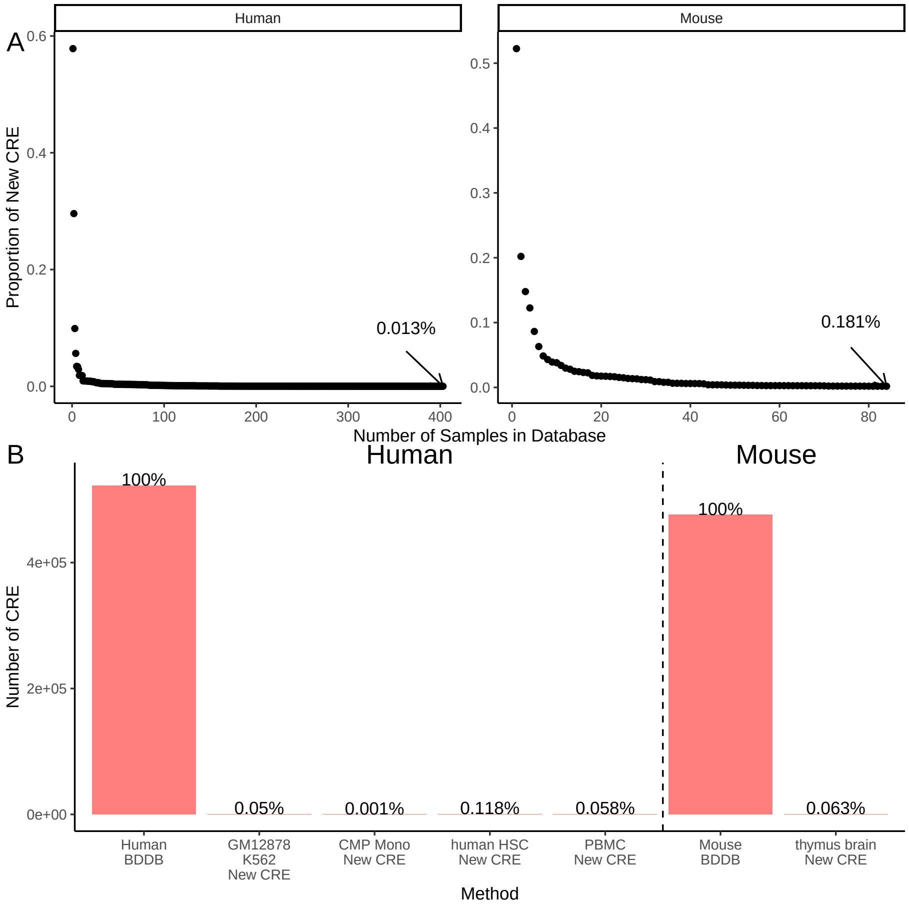

Figure S1: Saturation analysis of BDDB CRE lists. (A): As one increases the number of DNase-seq samples in the BDDB database, the proportion of new CREs contributed by adding a new sample gradually decreases. (B): The scATAC-seq datasets analyzed in this study would only add 0.0013%-0.118% new CREs to the precompiled CRE list in BDDB.

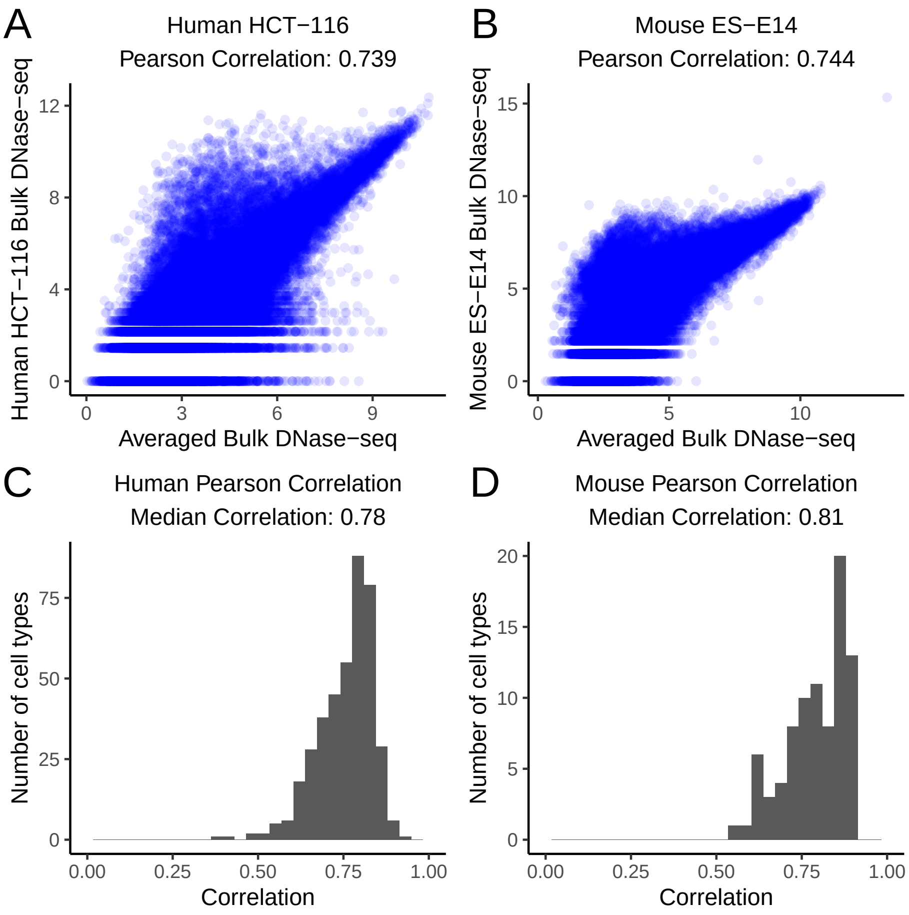

Figure S2: CRE activities explained by mean DNase-seq profile. (A): Scatter plot comparing CRE activities measured by bulk DNase-seq in human HCT-116 cell line versus CREs' baseline activities measured by the mean DNase-seq profile of all human BDDB samples. Each dot is a CRE. (B): Scatter plot comparing CRE activities measured by bulk DNase-seq in mouse ES-E14 cell line versus CREs' baseline activities measured by the mean DNase-seq profile of all mouse BDDB samples. (C): Analysis in (A) was repeated for all human DNase-seq samples in BDDB. Distribution of Pearson correlation coefficients between each DNase-seq sample and the mean DNase-seq profile is shown. (D): Analysis in (B) was repeated for all mouse DNase-seq samples in BDDB. Distribution of Pearson correlation coefficients between each DNase-seq sample and the mean DNase-seq profile is shown.

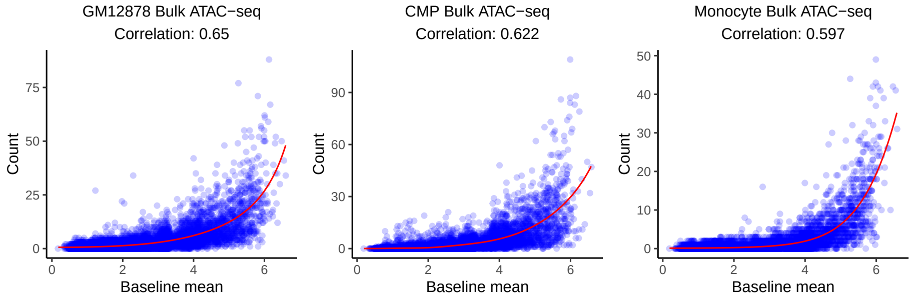

Figure S3: Normalization of bulk ATAC-seq and bulk DNase-seq data. The bulk ATAC-seq read counts in three different cell types (GM12878, CMP, Monocyte) versus baseline mean activities learned from BDDB bulk DNase-seq data are shown for low-variability CREs. Each blue dot is a low-variability CRE. In each plot, the red curve is the technical bias function fitted by SCATE.

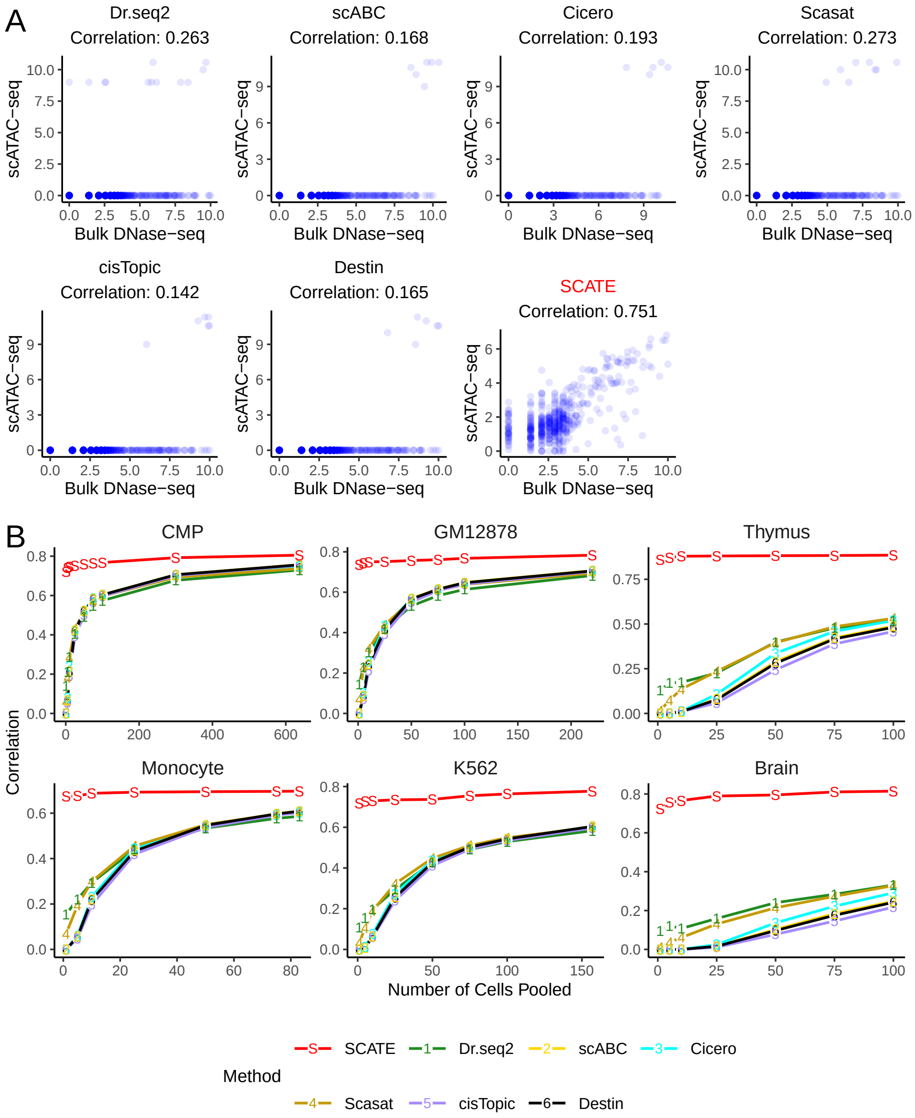

Figure S4: Comparison between SCATE and existing raw reads and binary methods based on the correlation between reconstructed and true CRE activities, similar to Figure 6. (A): Scatterplots showing true bulk CRE activities vs. CRE activities estimated by different methods in an analysis that pools 10 GM12878 cells. In this analysis, both activities are at log-scale. (B): The correlation between the scATAC-seq reconstructed and true bulk regulome for different methods. Each plot corresponds to a test cell type. In each plot, the correlation is shown as a function of the pooled cell number.

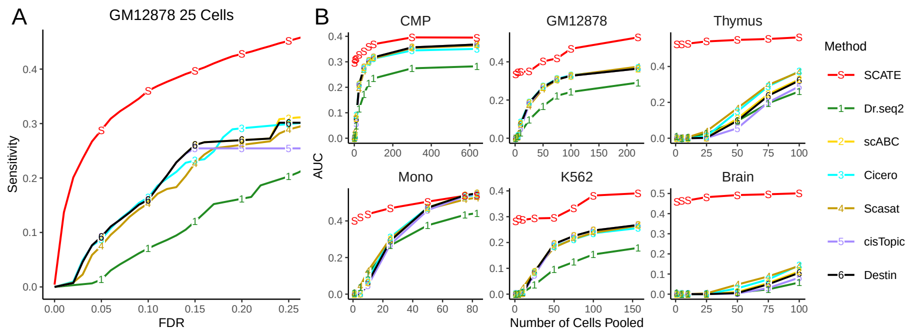

Figure S5: Comparison between SCATE and existing raw reads and binary methods based on the peak calling performance, similar to Figure 7. (A): The sensitivity versus FDR curve is shown for different peak calling methods in an analysis that pools 25 GM12878 cells. (B): The area under the sensitivity-FDR curve (AUC) is shown as a function of pooled cell number for different methods. Each plot corresponds to a different test cell type.

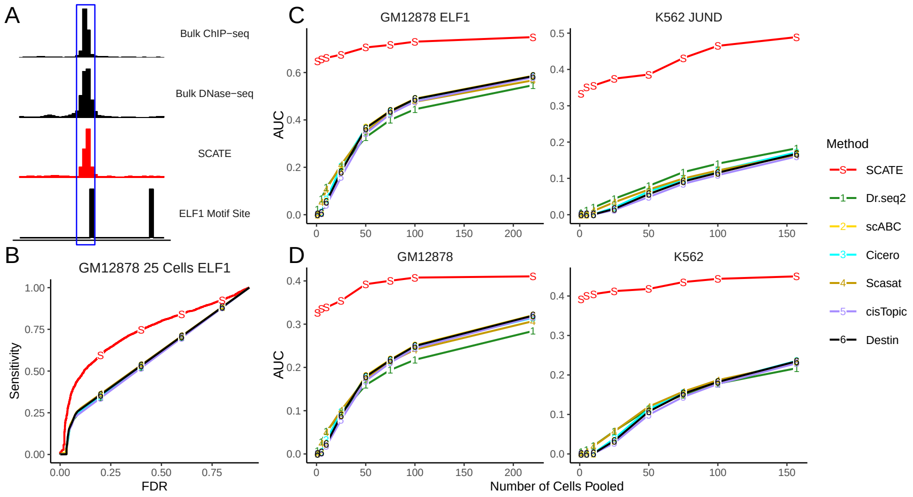

Figure S6: Comparison between SCATE and existing raw reads and binary methods based on TFBS prediction performance, similar to Figure 8. (A): The same as Figure 8A. (B): An example sensitivity versus FDR curve for comparing different methods for predicting ELF1 TFBSs in an analysis that pools 25 GM12878 cells. (C): Two examples (ELF1 in GM12878 and JUND in K562) that illustrate the method comparison across different cell numbers. In each example, analyses are performed by pooling different numbers of cells. The median AUC under the sensitivity-FDR curve from 10 independent cell samplings is shown as a function of pooled cell number. (D): The averaged AUC across all TFs is shown as a function of pooled cell number in GM12878 and K562 respectively.

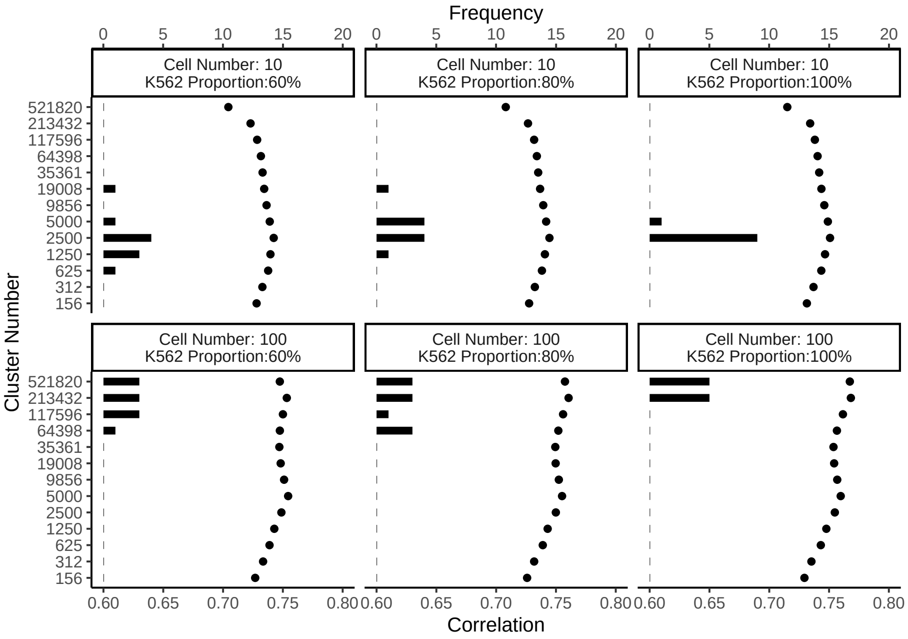

Figure S7: Adaptive tuning of analysis resolution in the presence of noise. K562 and GM12878 cells are mixed with different ratios, with K562 being the dominant cell type. The number of CRE clusters automatically chosen by SCATE via cross-validation (histogram) is compared with the optimal CRE cluster number determined by external information from the gold standard K562 bulk DNase-seq data (dots). Different plots correspond to different proportions of mixing GM12878 cells and K562 cells and pooled cell number. In each plot, the histogram shows the CRE cluster number chosen by SCATE in 10 independent cell samplings. The dots show the true correlation between the gold standard bulk DNase-seq signal and the SCATE-reconstructed scATAC-seq signal (both at log-scale) at each CRE cluster number, averaged across the 10 cell samplings. The dot with the highest correlation is the optimal cluster number chosen based on the bulk DNase-seq data.

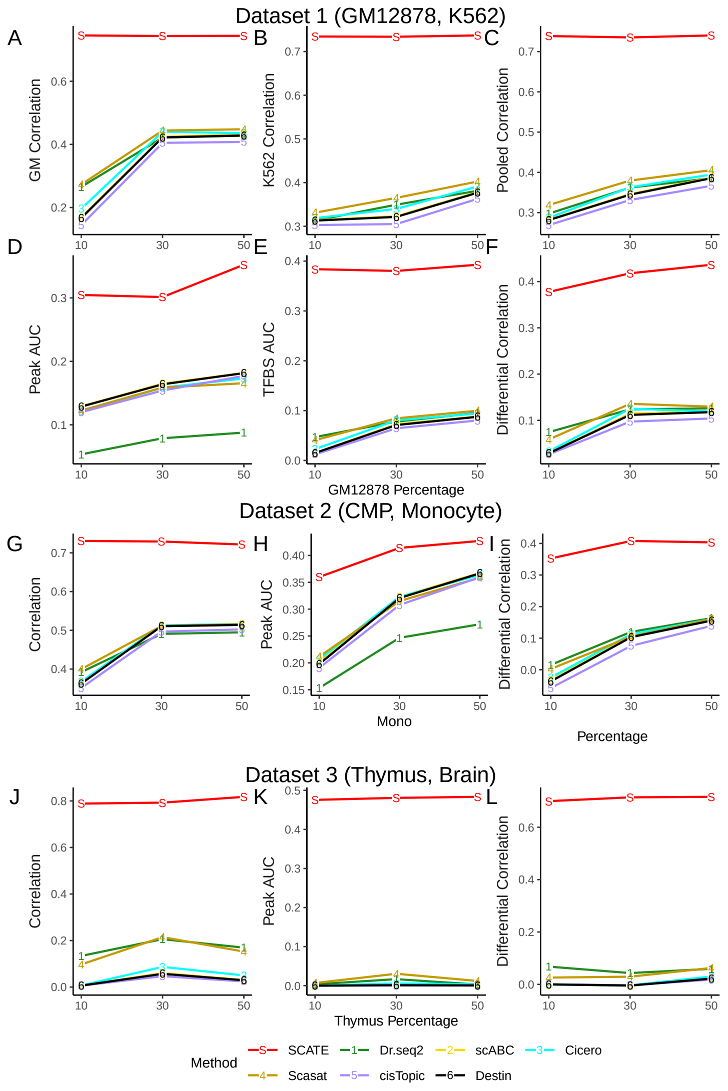

Figure S8: Comparison between SCATE and existing raw reads and binary methods in analyses of a heterogeneous cell population, similar to Figure 9. (A)-(C): The correlation between the scATAC-seq reconstructed and true bulk regulome in (A) GM12878, (B) K562, and (C) GM12878 and K562 combined for different methods is shown as a function of cell mixing proportion (GM12878 cell percentage). (D): The peak calling AUC (GM12878 and K562 combined) vs. cell mixing proportion. (E): The TFBS prediction AUC (GM12878 and K562 combined) vs. cell mixing proportion. (F): The correlation between the scATAC-seq reconstructed and true bulk differential log-CRE activities is shown as a function of cell mixing proportion. (G)-(I): Similar analyses in samples consisting of human CMP and monocyte cells, including (G) correlation between reconstructed and true bulk log-CRE activities, (H) peak calling AUC, and (I) correlation between predicted and true differential log-CRE activities. (J)-(L): Similar analyses in samples consisting of mouse thymus and brain cells, including (J) correlation between reconstructed and true bulk log-CRE activities, (K) peak calling AUC, and (L) correlation between predicted and true differential log-CRE activities.

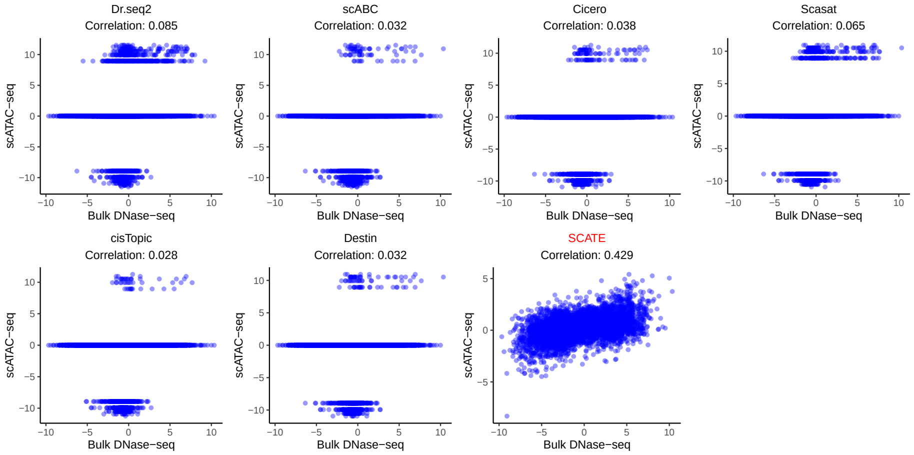

Figure S9: An example comparing SCATE with existing raw reads and binary methods for predicting differential CRE activities, similar to Figure 10. Scatterplots showing true bulk differential log-CRE activities vs. differential log-CRE activities estimated by different methods in an analysis of a synthetic sample consisting of 30 GM12878 and 70 K562 cells.

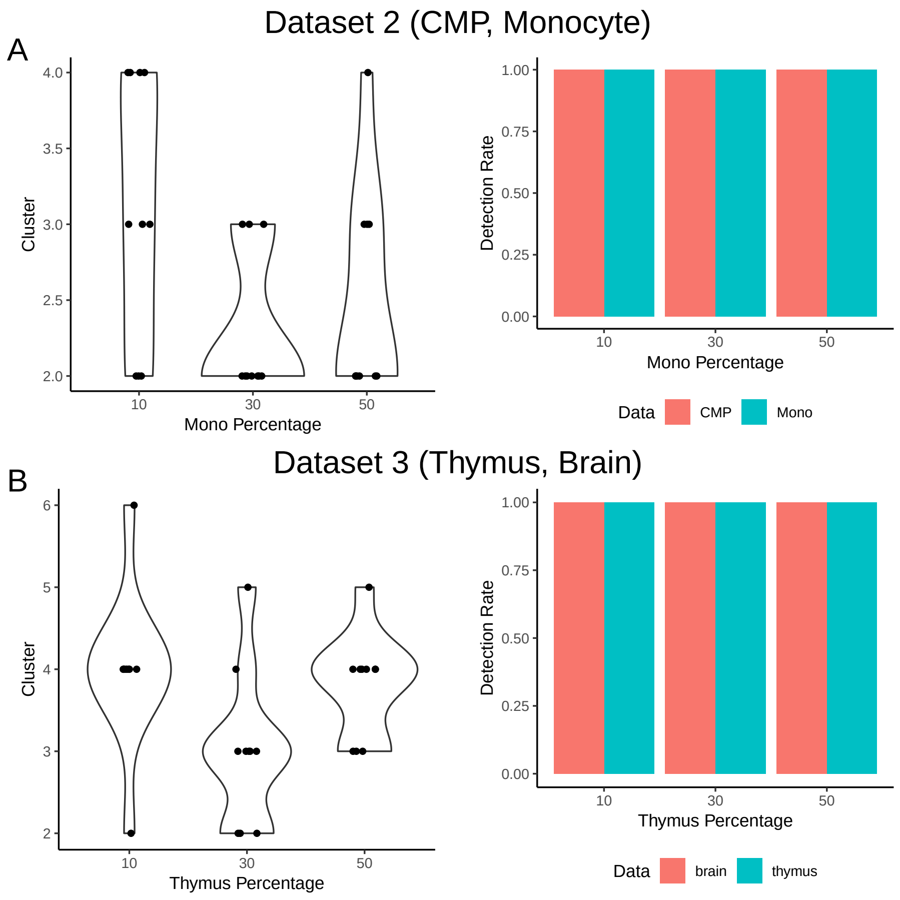

Figure S10: Analyses of a heterogeneous cell population created using (A) Dataset 2 and (B) Dataset 3. In each dataset, the left plot shows distribution of cell cluster numbers obtained by SCATE for synthetic samples with different cell mixing proportions. For each mixing proportion, 10 synthetic samples were created and analyzed. The right plot shows the frequency that each cell type is detected in the 10 synthetic samples at each cell mixing proportion.

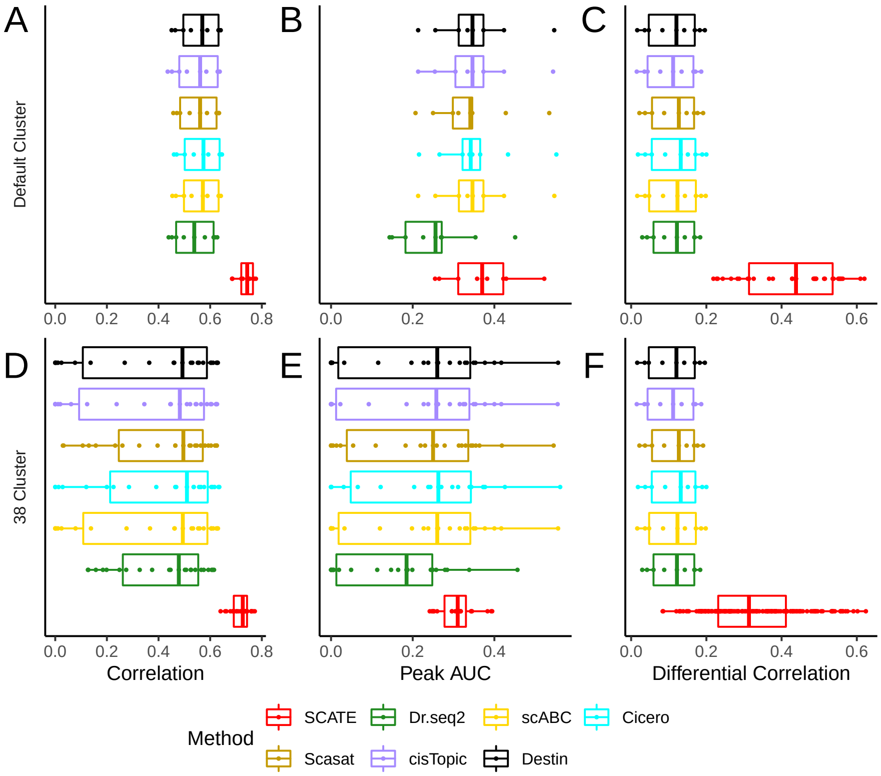

Figure S11: Comparison between SCATE and existing raw reads and binary methods in the analysis of human hematopoietic differentiation cell types, similar to Figure 11. (A)-(C): Regulome reconstruction performance of different methods in the default analysis, including (A) correlation between reconstructed and true bulk log-CRE activities, (B) peak calling AUC, and (C) correlation between predicted and true differential log-CRE activities. (D)-(F): Regulome reconstruction performance using user-specified cluster number (38 clusters), including (D) correlation between reconstructed and true bulk log-CRE activities, (E) peak calling AUC, and (F) correlation between predicted and true differential log-CRE activities.

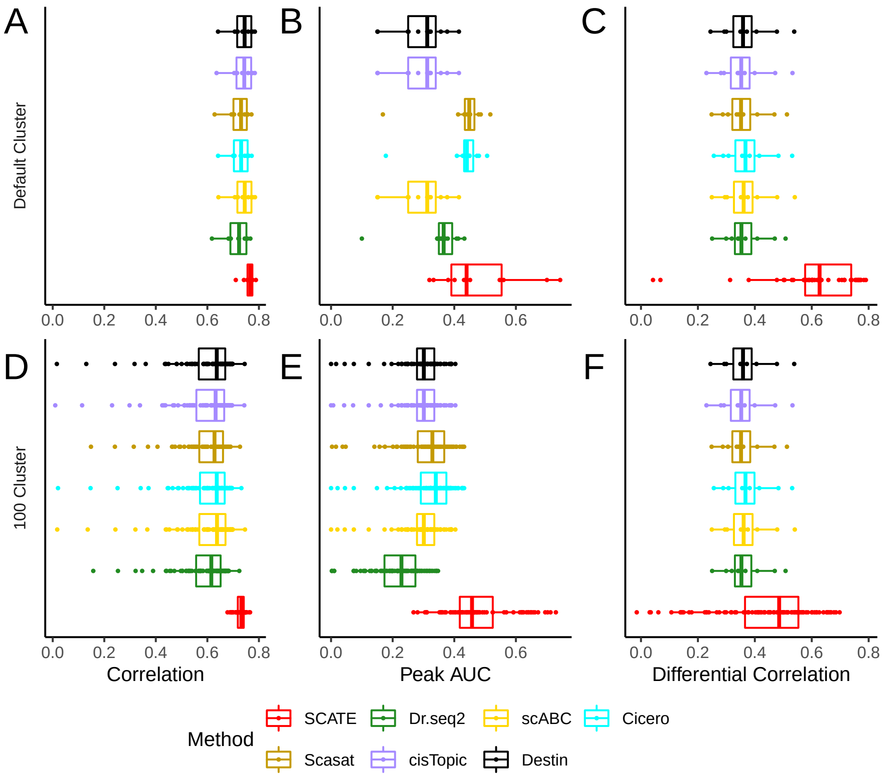

Figure S12: Comparison between SCATE and existing raw reads and binary methods in the analysis of human PBMC cell types, similar to Figure 12. (A)-(C): Regulome reconstruction performance of different methods in the default analysis, including (A) correlation between reconstructed and true bulk log-CRE activities, (B) peak calling AUC, and (C) correlation between predicted and true differential log-CRE activities. (D)-(F): Regulome reconstruction performance using user-specified cluster number (100 clusters), including (D) correlation between reconstructed and true bulk log-CRE activities, (E) peak calling AUC, and (F) correlation between predicted and true differential log-CRE activities.

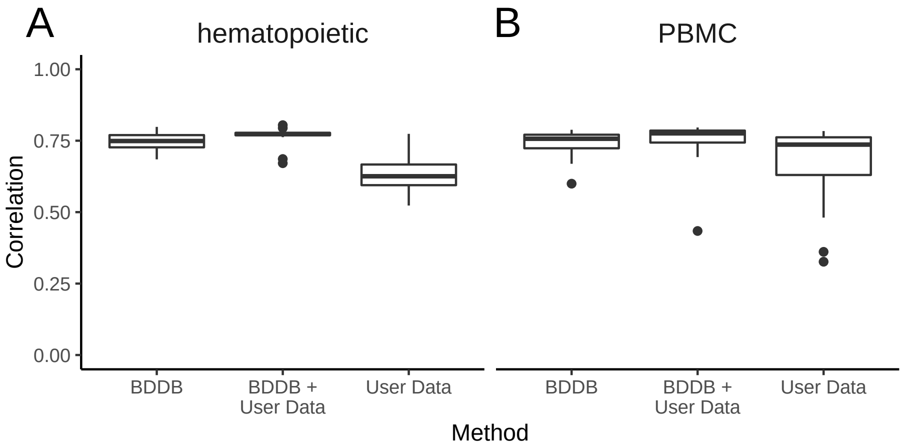

Figure S13: Comparison of SCATE(BDDB), SCATE(User Data) and SCATE(BDDB+User Data) in the analyses of two real datasets: (A) human hematopoietic differentiation cell types and (B) human PBMCs. Cells were first clustered using the default clustering algorithm in SCATE. CRE activities for each cell cluster were then inferred. For each method, the distribution of Pearson correlation between the reconstructed and true bulk log-CRE activities in all unambiguously annotated cell clusters is shown.

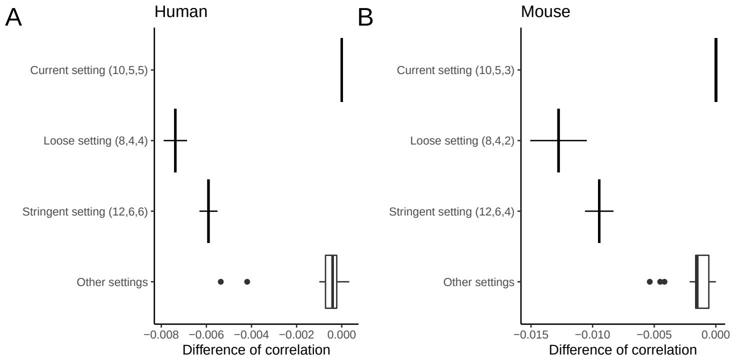

Figure S14: Exploration of preprocessing cutoffs for defining signal bins. BDDB bulk DNase-seq samples were partitioned into training and validation samples. Training samples were used to define signal bins. A subset of reads was sampled from each validation sample to mimic scATAC-seq data. SCATE using different cutoffs to define signal bins was applied to these mock scATAC-seq data to reconstruct signals. Pearson correlation between the SCATE output and the corresponding full bulk DNase-seq profile was computed. The difference in the correlation between each cutoff combination and the default cutoff combination was computed. The mean correlation difference across all validation samples is shown for the default cutoff combination, a looser cutoff combination, a more stringent cutoff combination, and other cutoff combinations in between. For the most loose and stringent settings, the mean correlation difference and standard error of the mean are shown. For the other settings, the distribution of the mean correlation difference is shown.

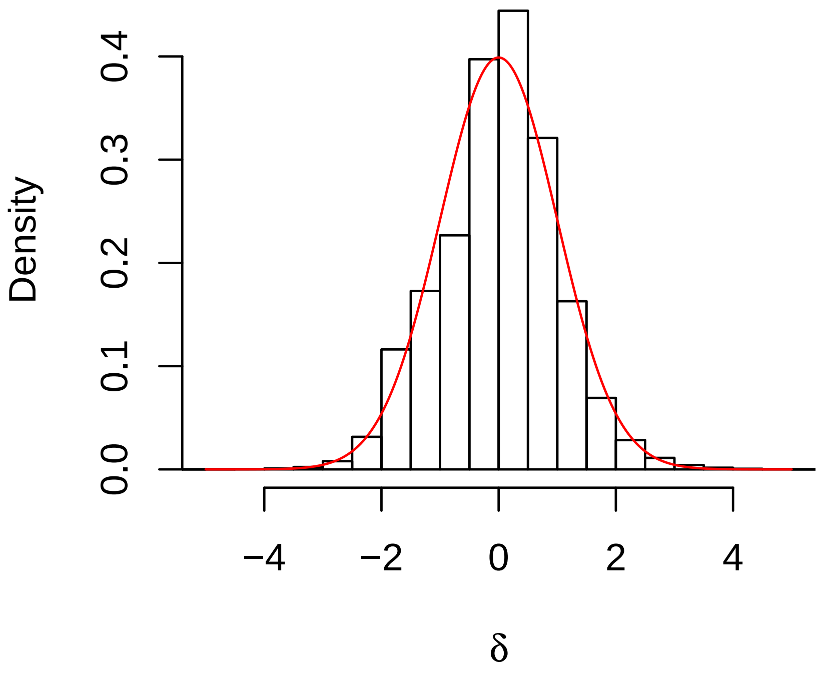

Figure S15: The empirical distribution (histogram) of the log-normalized read counts in human BDDB after standardization (i.e., subtract the mean and divide by SD of each CRE) can be fitted well with a normal distribution (red curve).
