# Supplementary material for: Single-cell ATAC-seq signal extraction and enhancement with SCATE
Source: Genome Biol. 2020 Jul 3;21:161. doi: 10.1186/s13059-020-02075-3 (PMC7333383; doi:10.1186/s13059-020-02075-3)
Supplement: Supplementary file 6 — Additional file 6 Supplementary note. Choice of preprocessing parameters for identifying signal bins. [file 13059_2020_2075_MOESM6_ESM.pdf]

Additional file 6 – choice of preprocessing parameters for identifying signal bins

In the data preprocessing for compiling CREs from the bulk DNase-seq data in BDDB, we define signal bins in each DNase-seq sample as follows: bin i is called a "signal bin" in sample j if (1) $y_{i,j} \geq 10$, (2) $\tilde{y}_{i,j} \geq 5$, and (3) $\tilde{y}_{i,j}$ is at least five times (three times for mouse) larger than the background signal defined as the mean of $\tilde{y}_{i,j}$s in the surrounding 100 kb region. The cutoffs for defining signal bins are used to filter out noisy genomic loci. Including such loci will increase computational burden. For example, we failed to run CRE clustering on our computer when we included all genomic bins in the analysis.

One can rewrite the criteria for defining signal bins as: (1) $y_{i,j} \geq a$, (2) $\tilde{y}_{i,j} \geq b$, and (3) $\tilde{y}_{i,j}$ is at least c times than the background signal. The default cutoff combinations in SCATE are $(a, b, c) = (10, 5, 5)$ for human and $(a, b, c) = (10, 5, 3)$ for mouse. We asked how different choices of (a, b, c) may affect the analysis performance. To this end, we explored different choices of cutoffs that were computationally feasible on our computer.

In order to ensure that data used to choose parameters are independent of the test data used to evaluate SCATE's performance on scATAC-seq, our exploration of cutoffs only used the bulk DNase-seq samples in BDDB. We did not use any scATAC-seq data for choosing these cutoffs. We also excluded all bulk DNase-seq samples whose cell types were involved in our test scATAC-seq data (e.g., human GM12878, K562, CMP, monocytes, etc.). In other words, the final test samples for benchmarking SCATE were not used in choosing our parameters.

The remaining BDDB DNase-seq samples were randomly partitioned into a training set and a validation set (For human: 357 samples were training samples and 18 samples were validation samples; for mouse: 61 samples were training samples and 7 samples were validation samples). The training samples were used to identify signal bins using the cutoff (a, b, c). SCATE was run by treating these training samples as BDDB. For each of the validation sample, 10,000 reads were randomly sampled from the corresponding bulk DNase-seq reads to simulate a scATAC-seq cell. SCATE was applied to these 10,000 reads to reconstruct CRE activities. The Pearson correlation between SCATE output and the true bulk DNase-seq profile was computed. We repeated this procedure for different cutoff combinations. For each cutoff combination (a, b, c), the difference in the Pearson correlation between the cutoff combination in question and the default cutoff combination is computed. A negative value of this correlation difference means decreased performance compared to the default cutoff combination. We conducted this analysis on all validation samples and computed the mean correlation difference across all validation samples for each cutoff combination.

For human, the default cutoffs used in our main article are $(a, b, c) = (10, 5, 5)$. Using a looser cutoff combination $(a, b, c) = (8, 4, 4)$ resulted in reduced signal reconstruction performance in validation samples (as measured by the mean correlation difference) (Additional file 2: Fig. S14). Similarly, using a more stringently cutoff combination $(a, b, c) = (12, 6, 6)$ also reduced the performance. We also explored cutoff combinations in between by setting $a = 8, 10, 12$, $b = 4, 5, 6$, and $c = 4, 5, 6$

and did not find any significant improvement in performance (Additional file 2: Fig. S14).

Similarly for mouse, the cutoffs used in our main article are $(a, b, c) = (10, 5, 3)$. Using a looser cutoff combination $(a, b, c) = (8, 4, 2)$ or a more stringent cutoff combination $(a, b, c) = (12, 6, 4)$ both reduced the signal reconstruction performance. Other cutoff combinations in between ($a = 8, 10, 12$, $b = 4, 5, 6$, and $c = 2, 3, 4$) also did not improve the performance (Additional file 2: Fig. S14).

These analyses explain why we used the cutoff values presented in the article.
