# Supplementary material for: Single-cell ATAC-seq signal extraction and enhancement with SCATE
Source: Genome Biol. 2020 Jul 3;21:161. doi: 10.1186/s13059-020-02075-3 (PMC7333383; doi:10.1186/s13059-020-02075-3)
Supplement: Supplementary file 7 — Additional file 7 Supplementary note. Derivation of moment estimators for mi and si. [file 13059_2020_2075_MOESM7_ESM.pdf]

Additional file 7 – derivation of moment estimators for m_i and s_i

Assume the read count $y_{i,j}$ for the i-th CRE and the j-th bulk DNase-seq sample is generated by a Poisson distribution:

$$y_{i,j} \sim Poisson(L_j \mu_{i,j})$$
$$\log(\mu_{i,j}) = m_i + s_i \delta_{i,j}$$

Further assume $\delta_{i,j} \sim N(0,1)$. According to the law of total expectation and total variance for conditional probability:

$$\begin{aligned}
\mathrm{E}\left(\frac{y_{i,j}}{L_j}\right) &= \frac{1}{L_j}\mathrm{E}[\mathrm{E}(y_{i,j}|\delta_{i,j})] \\
&= \mathrm{E}[\mu_{i,j}] \\
&= \mathrm{E}[e^{m_i+s_i\delta_{i,j}}] \\
&= e^{m_i}\mathrm{E}[e^{s_i\delta_{i,j}}] \\
&= e^{m_i+\frac{1}{2}s_i^2}
\end{aligned}$$

$$\begin{aligned}
\mathrm{E}\left(\frac{y_{i,j}}{L_j}\right)^2 &= \frac{1}{L_j^2}\mathrm{E}[\mathrm{E}(y_{i,j}^2|\delta_{i,j})] \\
&= \frac{1}{L_j^2}\mathrm{E}[Var(y_{i,j}|\delta_{i,j}) + \mathrm{E}(y_{i,j}|\delta_{i,j})^2] \\
&= \frac{1}{L_j^2}\mathrm{E}[L_j\mu_{i,j} + L_j^2\mu_{i,j}^2] \\
&= \frac{1}{L_j}\mathrm{E}[\mu_{i,j}] + \mathrm{E}[\mu_{i,j}^2] \\
&= \frac{1}{L_j}e^{m_i+\frac{1}{2}s_i^2} + \mathrm{E}[e^{2m_i+2s_i\delta_{i,j}}] \\
&= \frac{1}{L_j}e^{m_i+\frac{1}{2}s_i^2} + e^{2m_i+2s_i^2} \\
&= \frac{1}{L_j}e^{m_i+\frac{1}{2}s_i^2} + \left[e^{m_i+\frac{1}{2}s_i^2}\right]^2 e^{s_i^2} \\
&= \frac{1}{L_j}\mathrm{E}\left(\frac{y_{i,j}}{L_j}\right) + \left[\mathrm{E}\left(\frac{y_{i,j}}{L_j}\right)\right]^2 e^{s_i^2}
\end{aligned}$$

Thus we have:

$$e^{s_i^2} = \frac{\mathrm{E}\left(\frac{y_{i,j}}{L_j}\right)^2 - \frac{1}{L_j}\mathrm{E}\left(\frac{y_{i,j}}{L_j}\right)}{\left[\mathrm{E}\left(\frac{y_{i,j}}{L_j}\right)\right]^2}$$

and

$$s_i^2 = \log\left(\frac{\mathrm{E}\left(\frac{y_{i,j}}{L_j}\right)^2 - \frac{1}{L_j}\mathrm{E}\left(\frac{y_{i,j}}{L_j}\right)}{\left[\mathrm{E}\left(\frac{y_{i,j}}{L_j}\right)\right]^2}\right)$$

After replacing $\mathrm{E}\left(\frac{y_{i,j}}{L_j}\right)^2$ and $\mathrm{E}\left(\frac{y_{i,j}}{L_j}\right)$ by their sample estimates $\sum_j \left(\frac{y_{i,j}}{L_j}\right)^2/J$ and $\sum_j \left(\frac{y_{i,j}}{L_j}\right)/J$, respectively, we have:

$$\tilde{s}_i = \sqrt{\log\left(\frac{\sum_j (y_{i,j}/L_j)^2/J - \sum_j (y_{i,j}/L_j^2)/J}{(\sum_j (y_{i,j}/L_j)/J)^2}\right)}$$

Since

$$m_i \quad = \quad \log \mathrm{E}\left(\frac{y_{i,j}}{L_j}\right) - \frac{1}{2}s_i^2$$

We have

$$\tilde{m}_i = \log\left(\frac{\sum_j (y_{i,j}/L_j)}{J}\right) - \tilde{s}_i^2/2$$
